# Cytosolic TaGAPC2 Enhances Tolerance to Drought Stress in Transgenic Arabidopsis Plants

**DOI:** 10.3390/ijms21207499

**Published:** 2020-10-12

**Authors:** Lin Zhang, Hanwen Zhang, Shushen Yang

**Affiliations:** Life Sciences College, Northwest A&F University, Xianyang 712100, China; Adam_Panyuda@163.com (L.Z.); Z836361124@163.com (H.Z.)

**Keywords:** drought response, yeast two-hybrid system (Y2H), BiFC, TaGAPC, wheat

## Abstract

Drought is a major natural disaster that seriously affects agricultural production, especially for winter wheat in boreal China. As functional proteins, the functions and mechanisms of glyceraldehyde-3-phosphate dehydrogenase in cytoplasm (GAPCs) have remained little investigated in wheat subjected to adverse environmental conditions. In this study, we cloned and characterized a GAPC isoform *TaGAPC2* in wheat. Over-expression of *TaGApC2-6D* in Arabidopsis led to enhanced root length, reduced reactive oxygen species (ROS) production, and elevated drought tolerance. In addition, the dual-luciferase assays showed that TaWRKY28/33/40/47 could positively regulate the expression of *TaGApC2-6A* and *TaGApC2-6D*. Further results of the yeast two-hybrid system and bimolecular fluorescence complementation assay (BiFC) demonstrate that TaPLDδ, an enzyme producing phosphatidic acid (PA), could interact with *TaGAPC2-6D* in plants. These results demonstrate that *TaGAPC2* regulated by TaWRKY28/33/40/47 plays a crucial role in drought tolerance, which may influence the drought stress conditions via interaction with TaPLDδ. In conclusion, our results establish a new positive regulation mechanism of *TaGAPC2* that helps wheat fine-tune its drought response.

## 1. Introduction

Plants are constantly subject to various environmental stresses, including drought and salinity, which all lead to severe losses in crop yield. Under harsh stress conditions, the elaborate and complex signaling crosstalk with the environment has evolved and developed to perceive and translate these stress-signaling pathways, leading to protective responses [1,2]. All these adversities could greatly reduce crop yield and increase economic costs. A deeper physiological and genetic understanding of drought resistance is crucial for increasing food production.

Glyceraldehyde-3-P dehydrogenase (GAPDH) is widely considered to be a critical enzyme involved in the glycolytic and gluconeogenesis metabolic pathways by catalyzing the NAD-dependent conversion of glyceraldehyde-3-phosphate into 1,3-diphosphoglycerate. In all living organisms, glycolysis is an important metabolic pathway in carbohydrate metabolism [3]. In higher plants, there are four distinct isoforms of GAPDHs: GAPA, GAPB, glyceraldehyde-3-phosphate dehydrogenase in cytoplasm (GAPCs), and GAPCps. Phosphorylated and NADP-dependent GAPA and GAPB are involved in photosynthetic CO_2_ fixation in chloroplasts, phosphorylated and NADP-dependent GAPCs exist in the cytosol, and phosphorylated and NADP-dependent GAPCps exist in the non-green plastids. Nonphosphorylated and NADP-dependent NP-GAPDH exist in the cytosol [4,5,6].

NAD-dependent GAPDH consists of four identical subunits (C4). Generally, GAPDH is used as an internal control for the relative quantitation of gene expression because it is highly conserved in plants [7,8,9,10]. The gene structure and biochemical and functional properties of GAPDH have been investigated in mammals [11]. Recently, more and more studies have been carried out on the in vivo role of plant GAPDHs, especially in stress tolerance [12,13]. Many studies have also indicated that GAPDH proteins have many functions in addition to their roles in the glycolytic pathway [14,15,16]. In Arabidopsis, the relative expression level of *AtGApDH* was significantly increased in Arabidopsis roots after treatment with NaCl [17,18]. In the stable genetic Arabidopsis mutant plants (gapc1-1 and gapc2-1), the stomatal apertures and photosynthetic rate were significantly increased under drought stress, compared with the wild type [19]. The accumulation of GAPC1 has been found in the nuclei of root tip cells under cadmium treatment [20]. Under a low-concentration selenium environment, NAD-dependent GAPDHs are key regulatory factors that promote the normal growth of Arabidopsis seedlings [21]. The expression level and enzyme activity of GAPDH changes significantly during the immune response [22]. In rice, the expressions of *OsGApC1-3* were all induced by drought, salt, high temperature, ABA, and methyl viologen treatments. Further, the transgenic rice plants over-expressing *OsGApC3* are more resistant to salt stress compared with WT [4]. In maize, GAPC3 and GAPC4 proteins are synthesized under hypoxic conditions in the roots, which belong to the classic “anaerobic polypeptide”. Hypoxia can significantly increase the transcription level of the *GApC3/4* gene, while the *GApC1/2* gene maintains constitutive expression or decreases the transcription level. These studies all indicate that GAPC may play a role in the stress resistance of plants in some way.

Many previous studies, including our studies, have shown that the TaGAPC1 was up-regulated under certain abiotic stresses in *Chinese Spring Triticum aestivum* [23]. Thus, in order to investigate the function and stress response mechanism of *TaGApC2*, *TaGApC2-6A/6B/6D* were cloned in this study. Our results show that over-expression of *TaGApC2-6D* reduced the accumulation of reactive oxygen species (ROS) and promoted root growth in transgenic Arabidopsis plants under drought stress, which suggests that *TaGApC2-6D* plays an important role in enhancing drought tolerance by adjusting ROS content and promoting root length. Further, an osmotic-stressed full-length normalized cDNA library from *Chinese Spring* wheat was constructed and used to screen *TaGAPC2-6D*’s potential partners in a yeast two-hybrid system. This provides more detailed information for further investigation into the underlying mechanisms of *TaGApC2* involved in drought-related regulatory networks.

## 2. Results

### 2.1. Characterization of TaGApC2 and Bioinformatics Analysis

In our previous study, we identified six members of the *GApC* gene family in the wheat genome, among which *TaGAPDH3* (TraesCS6A02G213700.1), *TaGAPDH6* (TraesCS6B02G243700.1), and *TaGAPDH8* (TraesCS6D02G196300.2) are three copies of a gene, namely, *TaGAPC2-6A*, *TaGAPC2-6B*, and *TaGAPC2-6D* [24]. In the present study, three wheat *GApC2* genes—*TaGApC2-6A*, *TaGApC2-6B,* and *TaGApC2-6D*—were cloned from *Chinese spring* wheat and characterized. These three homologous genes share similar gene structures and are highly similar in terms of their amino acid sequences. Additionally, the numbers of exons and introns were also almost conserved within *TaGApC2* (Figure S1).

**Figure 1:**
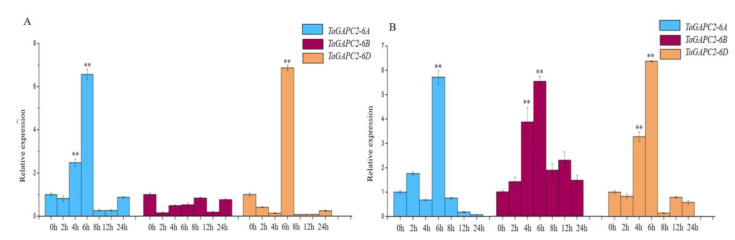
Transcription profiles of *TaGApC2* in *Chinese spring* wheat leaves under 20% PEG8000 (**A**) and 10 mM H_2_O_2_ (**B**). The *β-actin* gene was used as an internal reference. The vertical ordinate is the fold change; the horizontal ordinate is the treatment time. The data represent three independent experiments. The standard deviation (SD) is indicated at each point. Significant differences were assessed by one-sided paired *t*-tests (** *p* < 0.01).

To obtain more insights into the regulation mechanism of *TaGApC2* in *Chinese spring* wheat, the transcriptional expression patterns of *TaGApC2* genes, including *TaGApC2-6A*, *TaGApC2-6B,* and *TaGApC2-6D,* were examined at different time points (0, 2, 4, 6, 8, 12, and 24 h) after PEG and H_2_O_2_ treatment by quantitative real-time PCR (qRT-PCR). As shown in Figure 1, real-time PCR results show that the relative expression level of *TaGApC2-6A* and *TaGApC2-6D* increased over 2–8 h and reached the highest point. In addition, these genes were highly expressed in the roots, stems, and leaves of wheat plants (Figure S2).

### 2.2. Interaction between TaWRKY and TaGApC2-6D and TaGApC2-6A Promoters

**Figure 2:**
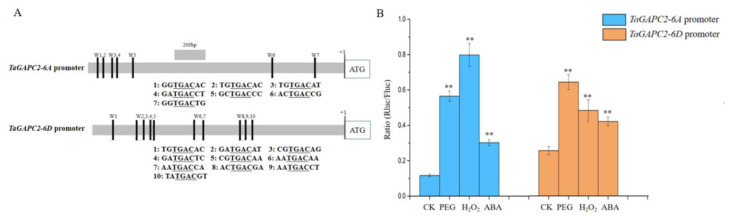
The *TaGApC2* promoter in *Chinese spring* wheat responds to abiotic stress in tobacco. (**A**) Schematic diagram of the distribution of the W-boxes in the *TaGApC2* promoter. (**B**) The Rluc/Fluc enzyme activity suggested that *TaGApC2* promoters are stress-inducible. The data represent three independent experiments. The standard deviation (SD) is indicated at each point. Significant differences were assessed by one-sided paired *t*-tests (** *p* < 0.01).

As shown in Figure 1 and Figure S2, *TaGApC2-6A* and *TaGApC2-6D* exhibited higher expression levels in different organizations and drought stress, showing that *TaGApC2-6A* and *TaGApC2-6D* might be the main effective genes for *TaGApC2* in wheat. Therefore, *TaGApC2-6A* and *TaGApC2-6D* were selected for further study. We firstly amplified and sequenced approximately 2.0 kb of the promoter regions upstream of the *TaGApC2-6A* and *TaGApC2-6D* ATG start codons from *Chinese spring* wheat, named *TaGApC2-6A*P and *TaGApC2-6D*P. Sequence analysis showed that *TaGApC2* promoter not only differs significantly in different wheat cultivars but also in different chromosomes of the same wheat cultivar (Figure S3). Furthermore, we also observed many cis-acting elements related to drought resistance in *TaGApC2-6A* and *TaGApC2-6D* promoters by bioinformatics analysis, including W-box elements; ABA-responsive elements (ABREs), MYB, and MYC binding sequences; and dehydration-responsive elements (DREs) (Table S1). *TaGApC2-6A*P and *TaGApC2-6D*P were fused to the Rluc reporter vector to detect promoter activity in tobacco leaves. The inducible activities of *TaGApC2-6A*P and *TaGApC2-6D*P under PEG, ABA, and H_2_O_2_ treatments were measured in transgenic tobacco by the Rluc/Fluc enzyme activity. The results show that *TaGApC2-6A*P and *TaGApC2-6D*P were stress-inducible promoters (Figure 2B).

**Figure 3:**
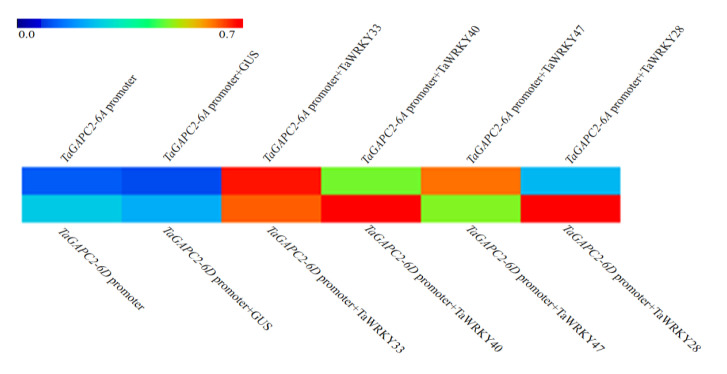
Rluc/Fluc value analysis of the interaction between TaWRKYs and *TaGApC2* promoters of *Chinese spring* wheat. *TaGApC2-6A* promoter and *TaGApC2-6D* promoter. The GUS effector was used as internal control. The data represent three independent experiments. The standard deviation (SD) is indicated at each point. Significant differences were assessed by one-sided paired *t*-tests.

We previously observed that the transcription factor TaWRKY28/33/40/47 could bind to the special W-box in *CW-TaGApC5* (*TaGApC2-6D* of wheat cv.*Changwu*) and *ZY-TaGApC5* (*TaGApC2-6D* of wheat cv.*Zhengyin*) promoter. The transcriptional expression patterns of several *TaWRKY* genes, including *TaWRKY28*, *33*, *40,* and *47,* were examined at different time points after PEG treatment by qRT-PCR [25]. *TaWRKY28*, *33*, *40,* and *47* all expressed a trend of increasing first and then decreasing under PEG stress, similar to that of *TaGApC2-6A* and *TaGApC2-6D* but before them. These results indicate that TaWRKY28, 33, 40, and 47 may also positively regulate the expression of *TaGApC2-6A* and *TaGApC2-6D* in *Chinese spring* wheat (Figure 1A). To further investigate whether TaWRKY28, 33, 40, and 47 could bind to *TaGApC2-6A* and *TaGApC2-6D* promoters in *Chinese spring* wheat, we firstly analyzed the type of W-box in *TaGApC2-6A* and *TaGApC2-6D* promoters in *Chinese spring* wheat. As shown in Figure 2A, these two promoters contain different types of W-box, including G/ATGACG/C/A bound by TaWRKY28, C/G/ATGACG bound by TaWRKY33, C/ATGACC bound by TaWRKY40, and C/ATGACC/G bound by TaWRKY47 [25]. To investigate the transactivation role of TaWRKY28/33/40/47 on the target *TaGApC2-6A* and *TaGApC2-6D* promoter in *Chinese spring* wheat, a dual-luciferase assay was conducted according to what was previously reported [26]. The promoter of *TaGApC2-6A* and *TaGApC2-6D* was cloned into the dual-luciferase report vector pC0390-RUC and transformed into Agrobacterium *GV3101* and then injected together with either pC0390-TaWRKY:GUS (effector) or pC0390-GUS empty vector (control) into tobacco leaves. The Dual-Luciferase^®^ Reporter Assay System (Promega, Madison, WI, USA) was used to assess the luciferase activity (RLuc/Fluc). The Rluc/Fluc ratio was significantly increased by simultaneous TaWRKY:GUS over-expression compared to GUS expression (Figure 3). These results indicate that TaWRKY28/33/40/47 are indeed upstream positive regulators of *TaGApC2-6A* and *TaGApC2-6D* genes in wheat cv. *Chinese spring*.

### 2.3. Morphological Changes and Drought Tolerance in Transgenic Arabidopsis Plants

**Figure 4:**
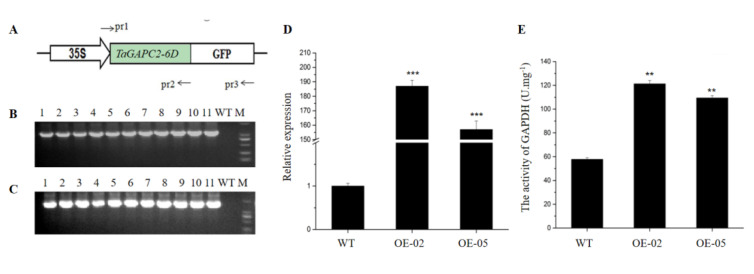
Detection of transgenic plants. (**A**) Diagram of the 35S:TaGAPC2-6D constructs. *TaGApC2-6D* CDS was fused with the GFP-coding region driven by a 35S promoter. The primers pr1, pr2, and pr3, used to analyze *TaGApC2-6D* and *TaGApCs-GFp* in transgenic Arabidopsis plants, are shown. (**B**) PCR analysis of *TaGApC2-6D* over-expressing transgenic Arabidopsis using primer pairs of pr1 and pr2. (**C**) PCR analysis of *TaGApC2-6D* over-expressing transgenic Arabidopsis using primer pairs of pr1 and pr3. (**D**) Quantitative real-time PCR (qRT-PCR) validation of *TaGApC2-6D* in the aboveground part of the three-week-old Arabidopsis plant. The *AtTubulin* gene was used as an internal reference (*** *p* < 0.001). (**E**) The enzyme activities of glyceraldehyde-3-P dehydrogenase (GAPDH) in the aboveground part of the three-week-old Arabidopsis plant (** *p* < 0.01).

To further understand the function of *TaGApC2*, we constructed transgenic Arabidopsis plants harboring p35S::*TaGApC2-6D*, expressing *TaGApC2-6D* under the control of the constitutive CaMV35S promoter. We selected putative transgenic lines harboring this construct on media containing hygromycin and confirmed them using PCR with gene-specific primers and qRT-PCR. The enzyme activity of GAPDH was also measured in WT and transgenic Arabidopsis plants (Figure 4). Two independent p35S::*TaGApC2-6D* transgenic lines (OE-02 and 05) were selected for further analysis.

**Figure 5:**
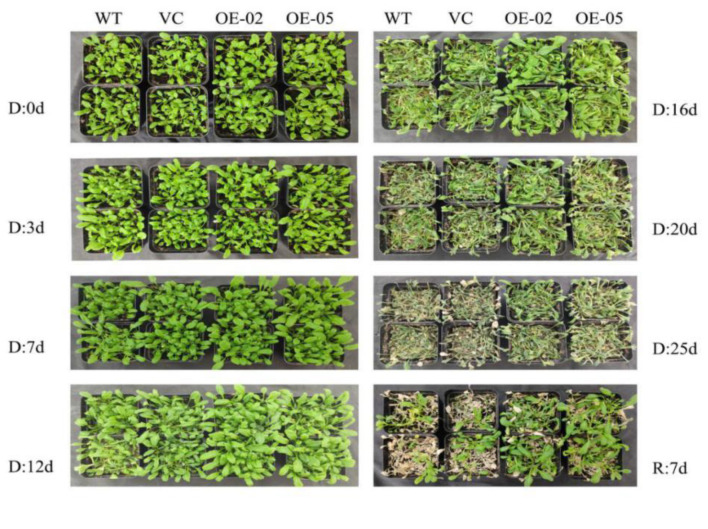
The phenotype of *TaGApC2-6D* over-expressing Arabidopsis plants after withholding water for 25 days. D, drought; R, re-watered; WT, wild type.

**Figure 6:**
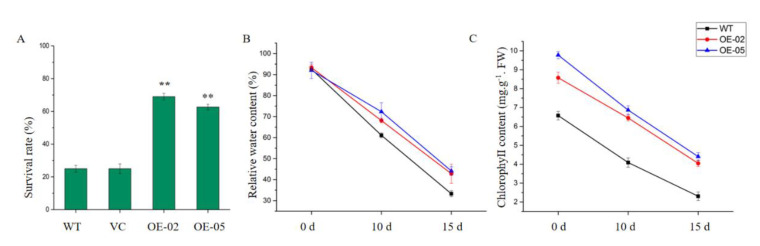
Physiological changes and tolerance assay in wild-type and transgenic plants under drought stress. (**A**) The survival rate of wild-type and transgenic Arabidopsis plants after drought treatment. At least 50 plants were counted and averaged for each line (** *p* < 0.01). (**B**) The relative water content (RWC) of wild-type and transgenic Arabidopsis plants after drought treatment. (**C**) The chlorophyll content of wild-type and transgenic Arabidopsis plants after drought treatment. The data represent three independent experiments. Asterisks indicate a significant difference between WT and transgenic Arabidopsis lines.

To investigate whether *TaGApC2-6D* over-expression was associated with drought stress tolerance, we subjected WT, vector control (VC), and transgenic three-week-old seedlings to 25 days of drought stress. At the early stage of treatment (7 days), all plants showed normal growth. At day 12 of treatment, the leaves of WT and the control line rapidly withered, whereas the growth of OE-02 and OE-05 plants was almost unaffected by drought stress. After 16 days of drought stress, both WT and the control line plants exhibited severe wilting. By contrast, the growth of OE-02 and OE-05 transgenic plants was better than that of WT. However, on the last day of drought treatment (day 25), all plants, including OE-02 and OE-05 transgenic plants, were severely wilted (Figure 5). Seven days after re-watering, 24% of the WT and 25% of the VC plants had survived, while the survival rates of the OE Arabidopsis were 68% and 62%, respectively (Figure 6A). Relative water content (RWC) and chlorophyll contents were relevant indicators for the measurement of drought tolerance. RWC and chlorophyll content were calculated in transgenic and WT Arabidopsis plants after 10 and 15 days of drought stress. As can be seen in Figure 6, the transgenic Arabidopsis lines had significantly higher RWC and chlorophyll contents than WT.

**Figure 7:**
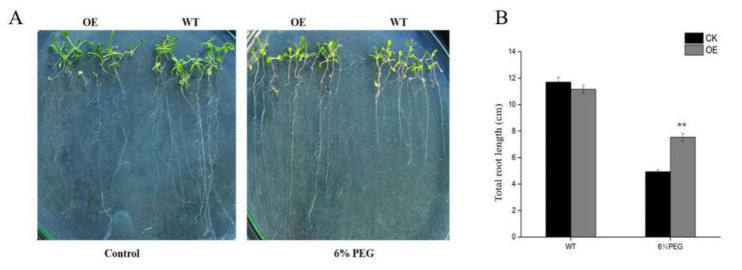
Total root lengths of transgenic Arabidopsis lines under mock drought stress. Phenotypes of WT and *TaGApC2-6D* transgenic Arabidopsis seedlings under 1/2 MS medium with or without 6% PEG8000. (**A**) Root lengths phenotype of WT and *TaGApC2-6D* transgenic Arabidopsis seedlings grown on 1/2 MS medium with or without 6% PEG8000 for seven days. (**B**) Root lengths of WT and *TaGApC2-6D* transgenic Arabidopsis seedlings grown on 1/2 MS medium with or without 6% PEG8000 for seven days. Data are means ± SD of three independent experiments, and asterisks indicate a significant difference between WT and transgenic *Arabidopsis* lines (** *p* < 0.01).

Transgenic and WT Arabidopsis seeds were grown on 1/2 MS medium for five days and then transferred to 1/2 MS medium containing 6% PEG8000. The OE-02 transgenic lines had a similar phenotype to WT plants under normal conditions. However, the total root length of the transgenic line was longer than that of WT plants under PEG8000 treatment after seven days. *TaGApC2-6D* more significantly promoted root growth in transgenic Arabidopsis plants than WT under PEG8000 treatment (Figure 7). The result suggests that the enhanced drought tolerance in *TaGApC2-6D* transgenic lines may be related to the longer root length.

### 2.4. Ectopic Expression of TaGApC2-6D Reduces ROS Accumulation under Drought Stress

**Figure 8:**
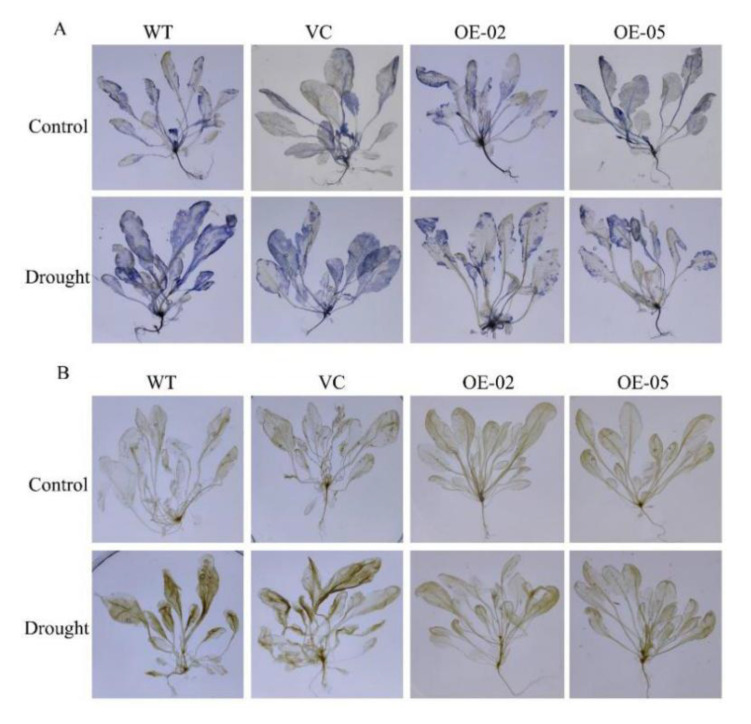
Changes in O_2_^−^ and H_2_O_2_ levels in wild-type (WT) and transgenic plants subjected to drought stress. Drought-stressed seedlings were incubated in nitroblue tetrazolium (NBT) or diaminobenzidine (DAB) solution. Blue staining indicates O_2_^−^ accumulation (**A**). Brown staining indicates H_2_O_2_ accumulation (**B**). Control, plants growing under normal conditions; drought, plants growing after drought stress treatment (12 days).

Since drought stress increases ROS production, we then investigated whether *TaGApC2-6D* over-expression in Arabidopsis would affect ROS accumulation under drought stress by examining O_2_^−^ and H_2_O_2_ accumulation using nitroblue tetrazolium (NBT) and diaminobenzidine (DAB) staining, respectively. The results obtained from histochemical staining indicate that transgenic Arabidopsis plants and WT generated different amounts of ROS (mainly O_2_^−^ and H_2_O_2_). As shown in Figure 8A, under normal conditions, little NBT staining was detected in the WT and VC, whereas after drought stress, clear NBT staining was detected in both lines. By contrast, lower NBT staining and content of O_2_^−^ were detected in the OE transgenic lines, even under drought stress (Figure 8A and Figure S4). In addition, lower signals of DAB staining and content of H_2_O_2_ were detected in the OE transgenic lines than in the WT and VC lines (Figure 8B and Figure S4).

**Figure 9:**
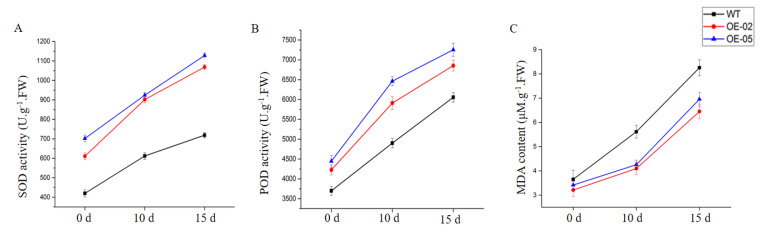
Effects of drought stress on superoxide dismutase (SOD) (**A**), peroxidase (POD) (**B**), and malondialdehyde (MDA) levels (**C**) in WT and transgenic plants after drought stress. The data represent three independent experiments.

Since malondialdehyde (MDA) content is an indicator of lipid peroxidation, MDA content was measured in the leaves of WT and transgenic Arabidopsis lines. After 15 days of drought treatment, the MDA content was significantly lower in the transgenic Arabidopsis lines, relative to WT (Figure 9C). Enzymatic antioxidants play a very significant role in scavenging harmful ROS that accumulates under stress. We further analyzed the superoxide dismutase (SOD) and peroxidase (POD) of these significant antioxidant enzymes in the leaves of the plants. These activities exhibited a similar trend in all lines after 10 and 15 days of drought stress (Figure 9A,B). After 10 and 15 days of drought stress, the activities of all antioxidant enzymes were higher in the *TaGApC2-6D* over-expression Arabidopsis lines than in WT. These results suggest that the enhanced drought tolerance in *TaGApC2-6D* transgenic lines is related to the reduced MDA content and the increased antioxidant enzyme activity.

### 2.5. Screening of Proteins Interacting with TaGApC2-6D by Yeast Two-Hybrid

**Figure 10:**
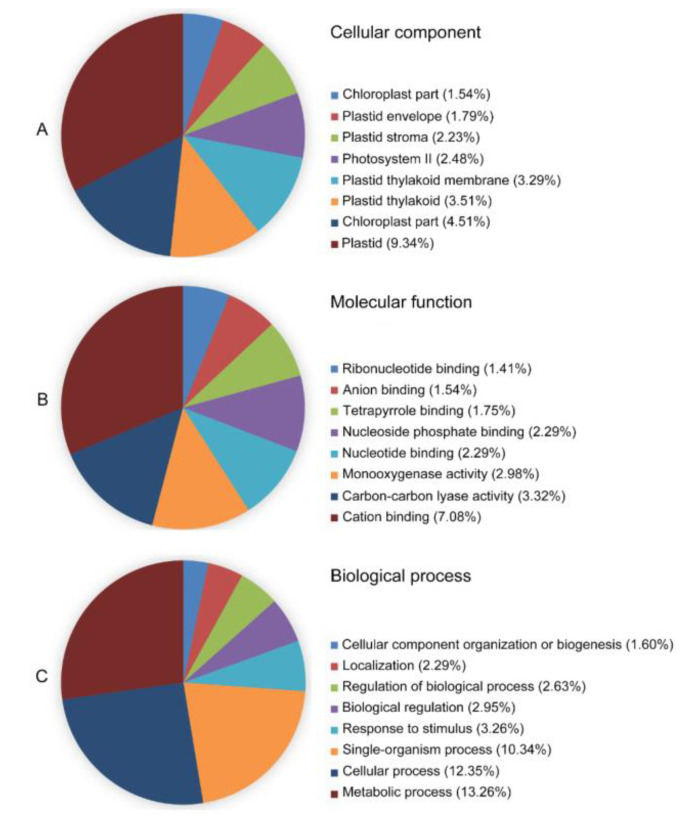
Functional annotation and classification of 804 *Triticum aestivum* annotated UniESTs using the BLAST2GO software. The three GO categories, cellular component (**A**), molecular function (**B**), and biological process (**C**), are presented.

**Figure 11:**
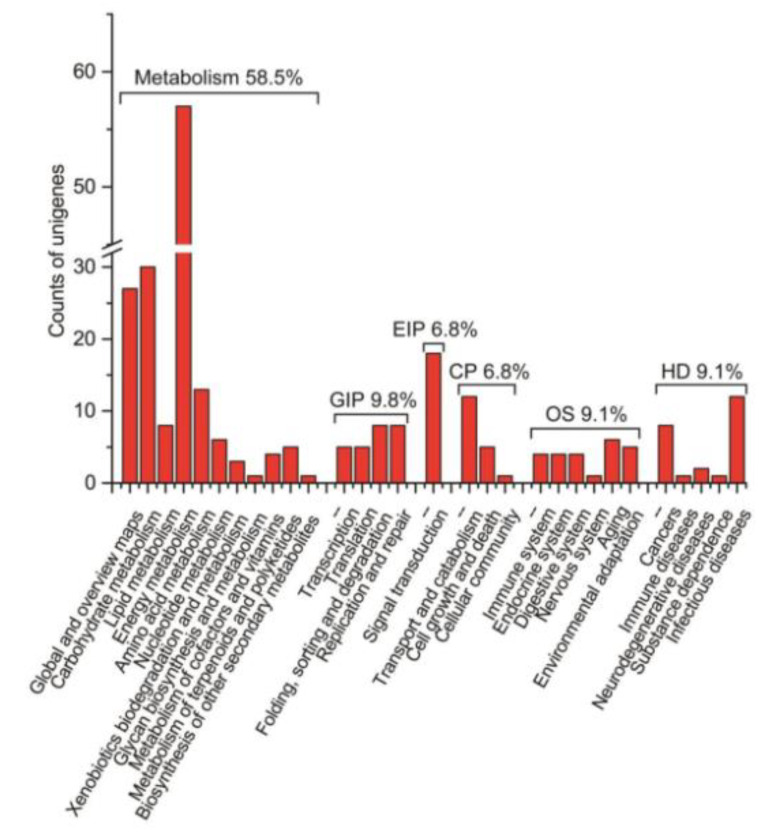
Functional annotation and classification of 804 Triticum aestivum annotated UniESTs using the KEGG pathway.

We firstly conducted an osmotic-stressed full-length normalized wheat cDNA library. The resultant library had a quantity of 5.30 × 10^5^ cfu/mL independent clones, and its titer was 3.62 × 10^7^ cfu/mL (Figure S5). To assess the quality of the library, a total of 1000 clones selected randomly from the cDNA library were sequenced, from which 804 high-quality expressed sequence tags (ESTs) from 963 raw sequences with an average length of 1048 bp were obtained. The 804 ESTs were clustered into 591 unigenes containing 64 contigs and 527 singletons. Based on GO annotation, the 579 of 591 unigenes were found in their homologous sequence and 330 different GO terms of cellular component (98), molecular function (71), and biological process (161) were obtained (Figure 10). Meanwhile, some transcription factors were hit, including transcription factor bHLH35, MADS-box transcription factor 5, transcription factor GLK2, and transcription factor MYB1R1-like, which were reported to play a key role in response to drought stress. The unigenes were subjected to analysis of biochemical pathways as described in the Kyoto Encyclopedia of Genes and Genomes (KEGG) database. The results show that 265 unigenes had their counterparts among 591 unigenes in the KEGG database, which relate to the six biological functions: metabolism, genetic information processing (GIP), environmental information processing (EIP), cellular processes (CP), organismal systems (OS), and human diseases. The metabolic pathways are the most abundant, making up 58.5% of the total (Figure 11). KEGG pathway analysis displayed that the cDNA library contained several plant stress response pathways, including the Ras signal pathway, MAPK signal pathway, TGF-beta signal pathway, HIF-1 signal pathway, and FoxO signaling pathway.

**Figure 12:**
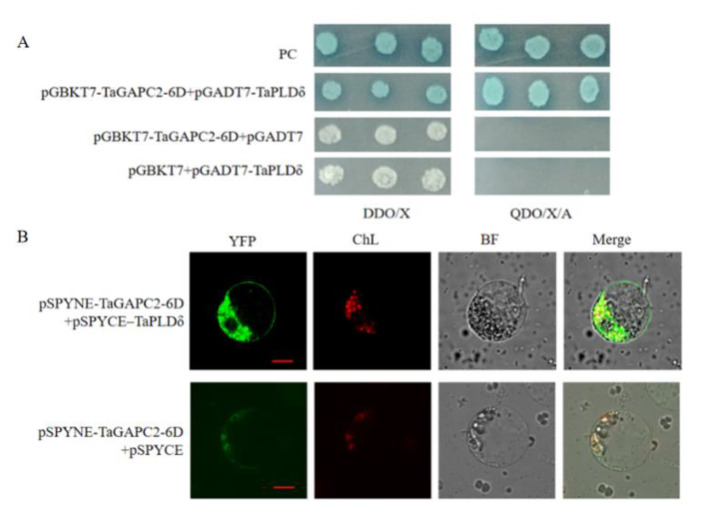
Identification of TaPLDδ interacting with TaGAPC2-6D. (**A**) Confirmation of true positive clones by small-scale yeast two-hybrid (Y2H) assay. SD/-Leu/-Trp/X-α-Gal (DDO/X), SD/-Leu/-Trp/X-α-Gal; SD/-Ade/-His/-Leu/-Trp/X-α-Gal/AbA (QDO/X/A), SD/-Ade/-His/-Leu/-Trp/X-α-Gal/AbA. PC indicates a positive control. (**B**) Bimolecular fluorescence complementation assay (BiFC) assay of the interaction between TaPLDδ and TaGAPC2-6D protein in tobacco leaf protoplasts. The pSPYNE-TaGAPC2-6D and pSPYCE-TaPLDδ constructs were co-infiltrated into tobacco by Agrobacterium. YFP fluorescence was detected by confocal laser scanning microscopy. Co-transformants of pSPYNE-TaGAPC2-6D and pSPYCE were used as negative controls. Scalebar = 100 μm.

To identify proteins that interact with *TaGAPC2-6D*, we used full-length *TaGAPC2-6D* as bait in the yeast two-hybrid (Y2H) system and screened a wheat osmotic-stressed cDNA library constructed from the prey vector pGADT7-Rec. To determine the specificity of interactions between *TaGAPC2-6D* and interacting proteins, the bait and positive library plasmid were co-transformed into Y2HGold strain pairwise. After exclusion of the false positives, 10 clones were identified as potential interaction partners of *TaGAPC2-6D* (Table S2). Among the putative binding partners of the *TaGAPC2-6D* protein, one was TaPLDδ, which could also interact with GAPCs in Arabidopsis. The bait and prey were again co-transformed into Y2HGold strain to confirm the interaction (Figure 12A).

To further verify the interaction between TaGAPC2-6D and TaPLDδ in plant cells, the bimolecular fluorescence complementation assay (BiFC) assay was performed in tobacco leaves. YFP fluorescence was reconstituted when pSPYCE–TaPLDδ and pSPYNE–TaGAPC2-6D were co-expressed. Recombinant vectors with corresponding non-fused pSPYCE empty vectors generated no fluorescence (Figure 12B).

## 3. Discussion

### 3.1. TaWRKY Positively Regulate TaGApC2 Gene Expression

In plants, there are many drought-responsive transcription factors that regulate the transcriptional expression of downstream genes by binding specific cis-acting elements of the promoter. WRKY transcription factors are one of the largest families of transcriptional regulators in plants, and are involved in the drought-related response [27]. In plants such as wheat [28], rice [29], brachypodium distachyon [30], maize [31], soybean [32], and cotton [33], a large number of WRKYs have been identified in recent years. In our previous study, we identified that TaWRKY28, TaWRKY33, TaWRKY40, and TaWRKY47 preferentially bind to specific W-box in *cw-TaGApC5* (*cw-TaGApC2-6D*) and *zy-TaGApC5* (*zy-TaGApC2-6D*) by Y1H and EMSA (Electrophoretic Mobility Shift Assay) analyses [25]. In *Chinese spring* wheat, the real-time PCR results indicate that the expression patterns of *TaGApC2-6A* and *TaGApC2-6D* were similar to *TaWRKY28/33/40/47* but lagged behind *TaWRKY28/33/40/47* under drought stress (Figure 1A). Thus, we speculated that TaWRKY28/33/40/47 may also be potential positive regulators of *TaGApC2-6A* and *TaGApC2-6D* expression in *Chinese spring* wheat. A tobacco co-transformation experiment demonstrated that TaWRKY28/33/40/47 indeed improves transcription levels of *TaGApC2-6A* and *TaGApC2-6D* (Figure 3). These results indicate that TaWRKY28/33/40/47 positively regulates *TaGApC2-6A* and *TaGApC2-6D* gene expression.

### 3.2. TaGApC2 Plays a Key Role in Drought Stress

*Arabidopsis thaliana* is often used as a model plant to study gene functions due to its short growth cycle, small nuclear genome, and high reproduction coefficient. Niu et al. constructed over-expression *TaDREB3* transgenic Arabidopsis plants to explore the function of the *TaDREB3* gene under drought, salt, and heat stresses [34]. Over-expression of *TaWRKY46* enhanced osmotic stress tolerance in transgenic *Arabidopsis thaliana* plants, which was mainly demonstrated by transgenic Arabidopsis plants forming a higher germination rate and longer root length [35]. In this study, a transgenic Arabidopsis over-expressing *TaGApC2-6D* was constructed through hygromycin screening and PCR identification, which provided experimental materials for studying *TaGApC2-6D* function. RWC, MDA, and chlorophyll content have been widely used to reflect drought tolerance in plants. The leaves of *TaGApC2-6D* overexpressing Arabidopsis plants exhibited higher survival rate, RWC, and chlorophyll content after drought treatment than WT plants (Figure 6). Further phenotypic analysis showed that *TaGApC2-6D* over-expressing Arabidopsis plants possessed longer roots at 6% concentrations of PEG (Figure 7).

In plants, ROS are the key cellular signals in response to multiple stresses, such as drought, wounding, and low and high temperatures. H_2_O_2_ and O_2_^−^ are the major and most stable type of ROS. Abiotic stress, such as drought stress, could cause excessive accumulation of ROS raising lipid peroxidation to interfere with normal physiological processes and ultimately leading to programmed cell death [36,37,38]. In order to explore the effect of *TaGApC2-6D* on antioxidant enzyme activity, the antioxidant enzyme activities (POD, SOD) were measured in wild-type and transgenic Arabidopsis under drought stress. The results show that the H_2_O_2_ and O_2_^−^ accumulation in transgenic Arabidopsis over-expressing *TaGApC2-6D* were less and the enzyme activities of POD and SOD were enhanced, compared with WT Arabidopsis (Figure 8 and Figure 9). The increased ROS clearance ability reduced cell membrane damage and enhanced drought resistance. All the results suggest that over-expression of *TaGApC2-6D* enhanced drought tolerance in Arabidopsis plants. A similar finding was also verified in other *TaGApDH* [39].

### 3.3. Interaction between TapLDδ and TaGApC2 May Contribute to Plant Drought Tolerance

To date, little is known about genes in wheat contributing to the strong adaptation in drought areas. The molecular mechanism for wheat’s adaptation to drought environments needs to be explained. It has been shown that full-length cDNA clones can be used to study the function of gene products [40]. In order to explore the biology of a plant response mechanism—genes such as those responsible for survival, dehydration avoidance, and development—a full-length cDNA library was constructed using wheat leaves from a drought condition. The high-quality-expression cDNA library can offer molecular resources for the analysis of genes involved in biology and for studying its protein function [41]. Our library storage was 5.30 × 10^5^ cfu/mL, and the titer was 3.62 × 10^7^ cfu/mL. The insert fragment size was greater than 0.75 kb, which proved that the cDNA library is of high quality and has a high amount of information. The normalized cDNA library can also increase the possibility of selecting the mRNA with low expression [42].

In this study, we have screened 10 proteins that potentially interact with *TaGAPC2-6D* from the osmotic-stressed cDNA library. A large number of studies have shown that GAPC could interact with some stress-related proteins to protect plants from damage under abiotic stress. In Arabidopsis plants, GAPC could interact with E3 ubiquitin ligase (SINAL7) to make it monoubiquitinated. Arabidopsis over-expressing *SINAL7* showed increased concentrations of hexose, sucrose, and plant biomass, accompanied by elevated drought resistance and delayed leaf senescence, which provided important clues revealing that GAPC is involved in the resistance mechanism [43]. The receptor protein kinase FERONIA (FERONIA, FER) interacts with GAPC to catalyze a key reaction in glycolysis and promote energy production [44]. In this work, a wheat PLDδ protein was selected as a target protein to verify potential interactions with *TaGAPC2* in yeast, and this interaction was further confirmed through bimolecular fluorescence complementation (Figure 12B). In the plant, PLD has been identified to respond to multiple stresses [45]. For example, *pLDα3* positively responds to hyperosmotic stress [46], *pLDα1* could respond to water stress by promoting ABA-regulated stomatal closure to reduce water loss, and *pLDα1* is also involved in the process by which lipid messenger PA produces peroxide products [47,48]. We propose that *TaGAPC2* may improve the drought tolerance of plants by interacting with PLDδ to promote ABA-regulated stomata closure. Our findings offer evidence of *TaGAPC2*’s roles in drought resistance on a molecular level. Further studies on whether *TaGAPC2* interacts with other proteins will be required to thoroughly understand *TaGAPC2*’s molecular function.

## 4. Materials and Methods

### 4.1. Plant Materials and Growth Conditions

Common wheat (*Triticum aestivum* L. cv. *Chinese Spring*) was used in this study. The seeds were surface-sterilized with 75% ethanol for 2 min and rinsed three times with sterile water. Seeds were placed on moistened filter paper in petri dishes for germination in darkness at 22 °C for 24 h. The sprout seedlings were grown in a chamber at 22 °C in a 16/8 h (light/dark) cycle with a growth medium using Murashige and Skoog (MS) liquid medium.

To construct an osmotic-stressed cDNA library, 10-day-old seedlings were immersed in 20% PEG8000 (polyethylene glycol) solution. The treated leaf samples were collected at 0, 1, 2, 6, 12, and 24 h after the treatment, immediately frozen in liquid nitrogen, and kept at −80 °C for RNA isolation.

*Arabidopsis thaliana* seeds were surface-sterilized with 70% (*v*/*v*) ethanol for 2 min followed by 4% (*v*/*v*) hypochlorite, cold-treated for three days at 4 °C for vernalization, and germinated on 1/2 MS medium agar plates. Plants were grown with a 16/8 h (light/dark) cycle photoperiod at 22 °C. Seedlings were transplanted to soil 10 days after germination.

### 4.2. Total RNA Extraction and Real-Time pCR Analysis

Total RNA was extracted using an RNAiso^TM^ Plus kit (TaKaRa, Dalian, China) and treated with RNase-free DNase I (TaKaRa, Dalian, China). The quality and purity of RNA were evaluated by Bioanalyzer 2100 and 1% agarose gel electrophoresis. First-strand cDNA was synthesized using a PrimeScript First-Strand cDNA Synthesis Kit (TaKaRa, Dalian, China). qRT-PCR was conducted using a Bio-Rad CFX96 system (BioRad, Hercules, CA, USA). The *β-actin* (AB181991.1) was used as the internal transcript level control for qRT-PCR using SYBR^®^ Premix Ex Taq™ (TaKaRa, Dalian, China). Detailed information on the primer sequences is provided in the supporting information (Table S3). All real-time PCR reactions were performed in triplicate to ensure reproducibility of the results.

### 4.3. Examination of Plant Stress Tolerance

The coding sequence of *TaGApC2-6D* was cloned into pCAMBIA1302 under the control of the CaMV 35S promoter, resulting in 35S::*TaGAPC2-6D* construct. This construct was used in transformation mediated by Agrobacterium to obtain transgenic Arabidopsis lines. Hygromycin-resistant Arabidopsis transformants carrying *TaGApC2-6D* were generated using the inflorescence infiltration method [49]. Transformed plants were cultured on 1/2 MS medium containing 30 µg/mL hygromycin in a day/night regime of 16/8 h under white light at 22 °C for 10 days and then transferred to soil.

The Enzyme-Linked Immunosorbent Assay (ELISA) Kits (Meimian Biotech, Lianyungang, China) was applied to detect the enzyme activities of GAPDH from the aboveground part of three-week-old Arabidopsis plants, which were homogenized with cold PBS. Then, the supernatants were collected after centrifugation and added to test wells for further reactions following the operating manual. The absorbance at 450 nm was measured using a microplate reader. All results were determined by the standard curve equation and normalized to the protein concentration of corresponding samples.

Homozygous T3 seeds of transgenic Arabidopsis lines were used for phenotypic analysis. Arabidopsis seeds were grown on 1/2 MS agar plates that were routinely kept for three days in darkness at 4 °C to break dormancy and then transferred to a tissue culture room at 22 °C for five days. For drought treatment, 5-day-old seedlings were then transferred to 1/2 MS agar plates containing 6% PEG8000 for seven days. The total root lengths of the Arabidopsis plants were measured [50]. To analyze the drought tolerance in transgenic plants, the seedlings cultivated with soil in pots for three weeks at uniform developmental stages were subjected to drought stress treatment by withholding water. After being treated for 25 days, the plants were rewatered for seven days and then the survival rates of the different plant lines were recorded. Three biological experiments were performed, and at least 50 plants were used for the calculation.

H_2_O_2_ content detection kit and O_2_^−^ content detection kit (Solarbio, Beijing, China) were used to detect H_2_O_2_ content and O_2_^−^ content. O_2_^−^ and H_2_O_2_ accumulation was measured as previously described [26]. Determination of the RWC and the chlorophyll content of Arabidopsis leaves after drought stress were performed as previously described [26]. The MDA content and SOD and POD activity were determined as described [26].

### 4.4. Construction of the cDNA Library

The cDNA library was constructed using the Make Your Own “Mate&Plate™” Library System User Manual (Clontech, Mountain View, CA, USA) according to the manufacturer’s instruction. The first single-stranded cDNA (ss cDNA) were synthesized by 1 μg of total RNA using CDS III/3′ PCR primer and Moloney Murine Leukemia Virus (MMLV) Reverse Transcriptase. The double-stranded cDNA (dscDNA) was acquired by 2 μL of sscDNA product using Advantage 2 Polymerase Mix (Clontech) via long-distance PCR (LD-PCR). Then, the dscDNA products were digested with proteinase K, treated with DSN solution, and purified with a CHROMA SPINTM TE-400 column (Clontech). The purified dscDNA was ligated with pGADT7-Rec vector (*SmaI*-lineared) via homologous recombination using Clone Express^TM^ II One Step Cloning Kit (Clontech). The products were transformed into *Escherichia coli DH5α* strains, which were stored in 50% glycerol at −80 °C.

The transformed strains were diluted using LB medium (Amp) with the ratio 1:105 and then spread on 15 cm agar LB plates (Amp/IPTG/X-gal) and incubated at 37 °C for 12 h. The library titer and clone capacity were calculated by counting the clones from the plates. The library titer (cfu/mL) = colonies on LB/IPTG/X-gal × dilution factor ÷ volume (mL) plated. Colonies were randomly selected to identify the size of the inserted sequences and amplified with T7 SP and 3′AD primers. The library plasmid was isolated by High Pure Maxi Plasmid Kit (TianGen Biotech, Beijing, China).

### 4.5. Sequencing and Analysis of Expressed Sequence Tags (ESTs)

The transformed bacteria were randomly selected and inserted, and cDNA was sequenced from the 5′ end. The Codon Code Aligner program was used to remove vector sequences and assemble EST sequences into contigs. ESTs greater than 200 bp in length were regarded as high-quality ESTs after removal of empty ESTs and vector ESTs. The PHRED program was used for base calling and quality assessment [51]. The EST sequences were clustered and assembled into successive consensus sequences (contigs) using the Codon Code Aligner program, which had high stringency parameters of minimum percent identity of 99%, minimum overlap length of 50, and default parameters for the rest. A cluster containing only one sequence was classified as a singleton. All similarity searches were batch-executed locally using BlastN, BlastX, and TBlastX tools [52,53]. The Blast2go software package was used for GO annotations. By comparing the results with the Kyoto Encyclopedia of Genes and Genomes (KEGG, ftp://ftp.genome.jp/pub/kegg/) database, the functions of most unigenes were obtained. Finally, the sequences were classified as functional categories using the GO database [54].

### 4.6. Yeast Two-Hybrid Screen

To identify proteins that interact with TaGAPC2-6D, we screened a wheat drought-treated full-length normalized cDNA library in the yeast two-hybrid system. A fragment of *TaGApC2-6D* with open reading frame (ORF) was amplified from *Triticum aestivum* L. cv. *Chinese Spring* cDNAs using primers and subsequently cloned into the pGBKT7 vector (Clontech) as baits. The bait construct was transformed into the yeast strain Y2HGold and tested for auto-activation of MEL1 and AUR1-C reporter genes. The library plasmid was used as the prey. The prey vectors were transformed into the yeast strains Y2HGold containing bait vector and without auto-activation according to the manufacturer protocol. The transform products were plated onto synthetic dropout (SD) medium without leucine (Leu), tryptophan (Trp), and histidine (His) (TDO). Then, all the colonies that grew on TDO were screened and patched out onto higher stringency SD medium without Leu, Trp, His, and adenine (Ade) (QDO), QDO containing Aureobasidin A (AbA) (QDO/A), and QDO containing AbA and X-α-gal (X) (QDO/A/X), in turn. All plasmids of the colonies that grew on QDO/A/X were isolated using the yeast plasmid mini preparation kit (Beyotime, Shanghai, China), and the plasmids inserts were PCR-amplified using the universal primer on the pGBKT7 vector. To confirm positive interactions, the baits and positive library plasmids were co-transformed into Y2HGold stain according to the manufacturer protocol.

### 4.7. Bimolecular Fluorescence Complementation (BiFC) Assay

BiFC assay was performed in *Nicotiana benthamiana* leaves. The ORFs of *TapLDδ* and *TaGApC2-6D* without termination codons were recombined with C-terminal part of YFP in the pSPYCE-35S/pUC-SPYCE vector and N-terminal part of YFP in the pSPYNE-35S/pUC-SPYNE vector, respectively. The pSPYCE-TaPLDδ and pSPYNE-*TaGAPC2-6D* were co-transformed into *Nicotiana benthamiana* leaves with corresponding empty vectors as negative controls. The *Nicotiana benthamiana* leaves’ protoplasts were observed for fluorescence at 48 h after transformation using a confocal laser scanning microscope (Andor, Belfast, UK).

## 5. Conclusions

Taken together, the results presented in this study provide evidence that the transcription factor TaWRKY positively regulates the expression level of the *TaGApC2* gene by specially binding to its promoter in *Chinese spring* wheat. Over-expression of *TaGApC2* in Arabidopsis led to lower ROS production, longer root length, and higher drought tolerance under drought stress. *TaGAPC2* can interact with TaPLDδ, and it plays a vital role in plant drought tolerance. Our research provides a better understanding of the molecular mechanism of *TaGAPC2* in response to drought stress and offers a promising strategy to increase crop stress tolerance using bioengineering technology.

## Supplementary Materials

Supplementary materials can be found at https://www.mdpi.com/1422-0067/21/20/7499/s1.

## Abbreviations

GAPDH	Glyceraldehyde-3-phosphate dehydrogenase
GAPC	Glyceraldehyde-3-phosphate dehydrogenase in cytoplasm
qRT-PCR	Quantitative real-time PCR
Y2H	Yeast two-hybrid
BiFC	Bimolecular fluorescence complementation assay
SDO	SD/-Trp
SDO/X	SD/-Trp/X-α-Gal
SDO/X/A	SD/-Trp/X-α-Gal/AbA
DDO/X/A	SD/-Leu/-Trp/X-α-Gal/AbA
QDO/X/A	SD/-Ade/-His/-Leu/-Trp/X-α-Gal/AbA
ROS	Reactive oxygen species
