# Supplementary material for: Cytosolic TaGAPC2 Enhances Tolerance to Drought Stress in Transgenic Arabidopsis Plants
Source: Int J Mol Sci. 2020 Oct 12;21(20):7499. doi: 10.3390/ijms21207499 (PMC7590034; doi:10.3390/ijms21207499)
Supplement: Supplementary file 1 [file ijms-21-07499-s001.pdf]

## Supporting Information

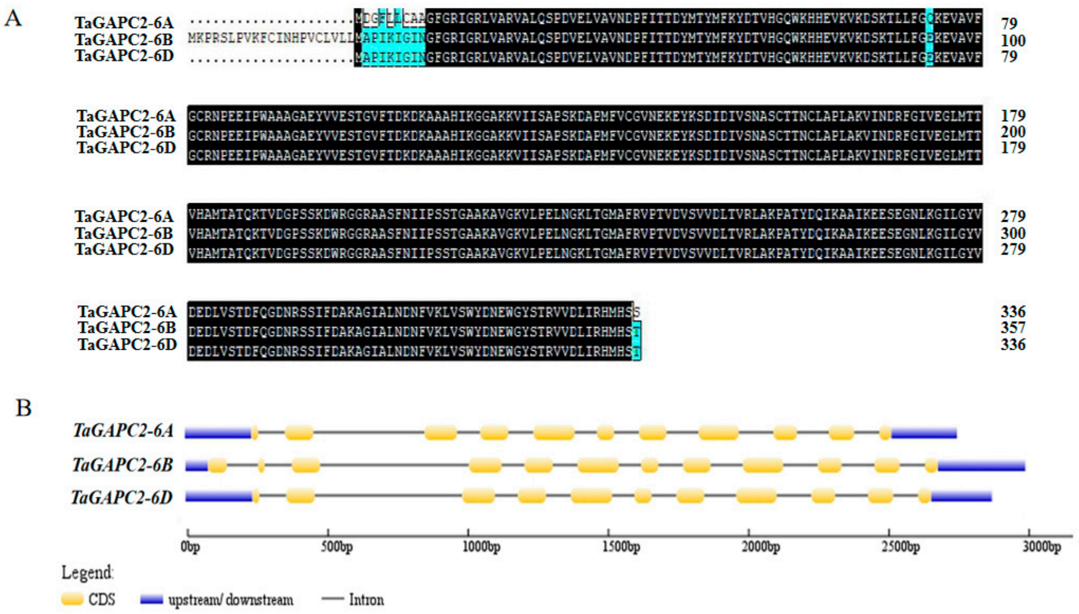


**Figure S1.** Sequence analysis. (A) Alignment of the Amino acid sequence of TaGAPC2. The identical and 75% amino acid sequence similarity are separately indicated by mazarine and green color. (B) Exons, introns, upstream/downstream are indicated by yellow boxes, black horizontal lines and blue boxes, respectively.

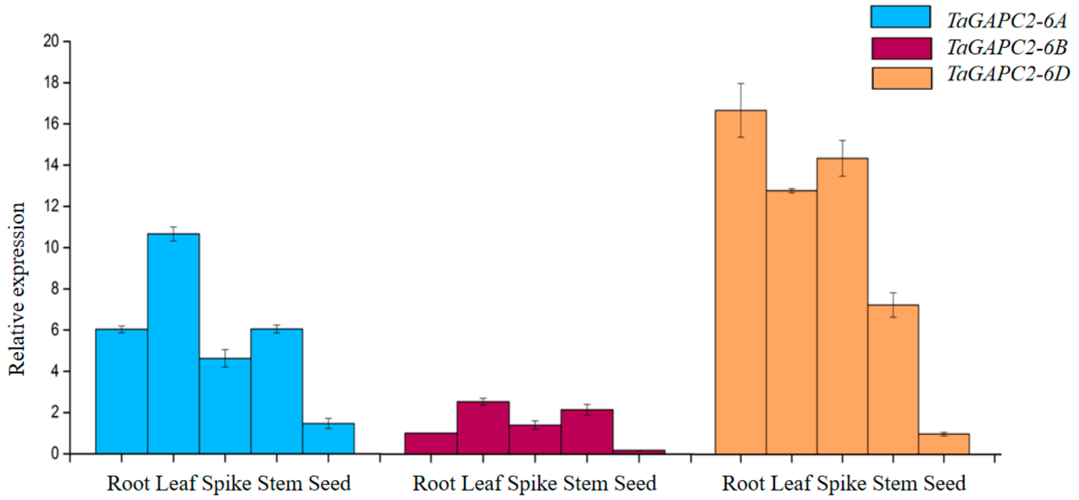


**Figure** S2. Organ expression assay of TaGAPC2 in different wheat organs. The indicated values are the average of three independent experiments. The standard deviation (SD) is indicated at each point.

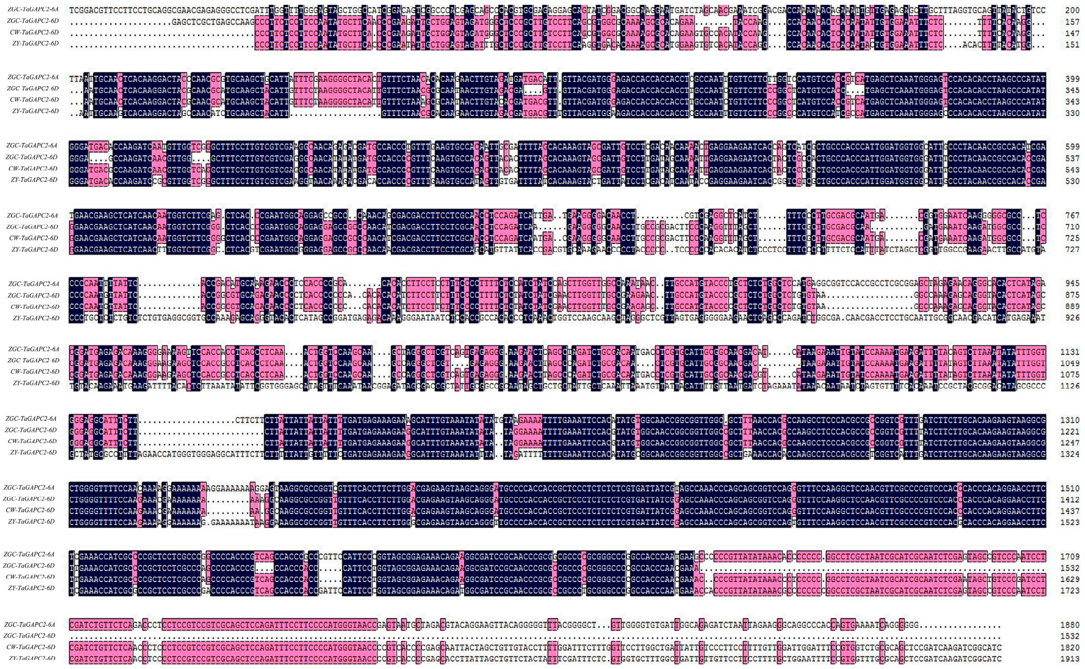

**Figure S3.** Promoter sequence alignment of *TaGAPC2* in wheat. *ZGC-TaGAPC2-6A*: The *TaGAPC2-6A* promoter in *Chinese spring* wheat; *ZGC-TaGAPC2-6D*: The *TaGAPC2-6D* promoter in *Chinese spring* wheat; *CW-TaGAPC2-6D*: The *TaGAPC2-6D* promoter in *Chang Wu* wheat; *ZY-TaGAPC2-6D*: The *TaGAPC2-6D* promoter in *Zheng Yin* wheat. The identical and 75% amino acid sequence similarity are separately indicated by mazarine and pink color.

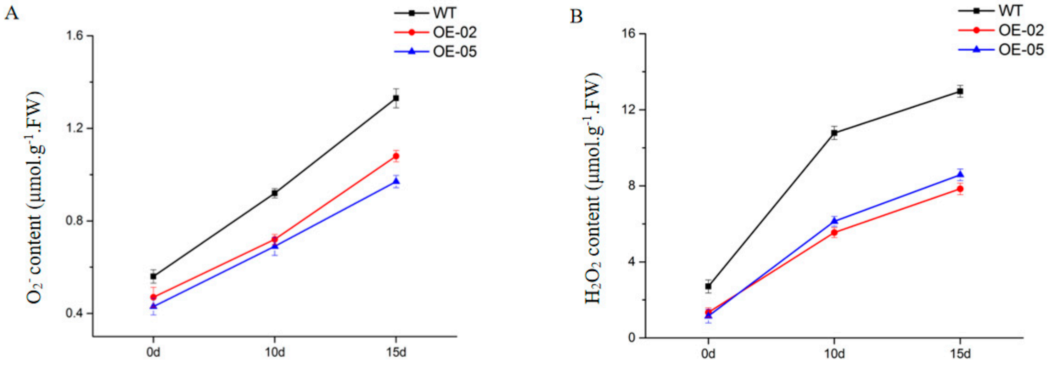


**Figure S4.** The contents of $O_2^-$ (A) and $H_2O_2$ (B) in WT and transgenic plants after drought stress.The datas are representative of three independent experiment.

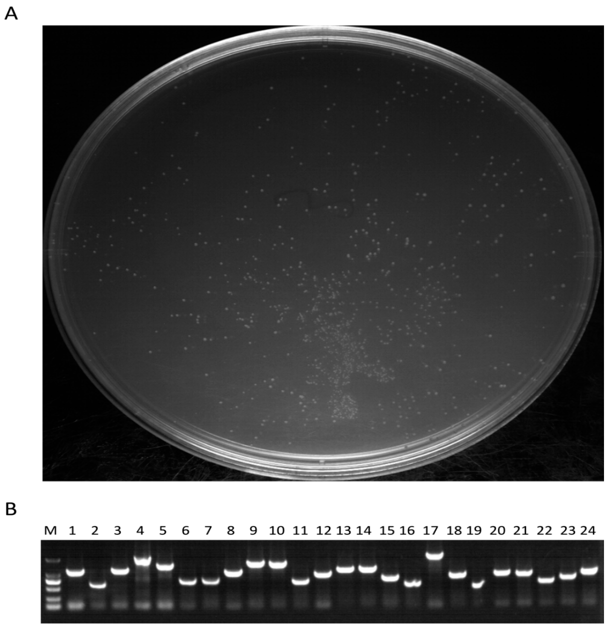


**Figure S5.** Quality of cDNA library (Yeast). (A) Measurement result of the library titer. (B) Detecting of the insert fragments.

**Table S1.** Putative cis-acting elements in the TaGAPC2 promoters.

| Gene Element | ABRE | CBFHV | DRE/CRT | GARE | LTRE | MYB | MYC | W-box |
|---|---|---|---|---|---|---|---|---|
| *TaGAPC2-6A* Promoter | 3 | 2 | 3 | 1 | 2 | 10 | 4 | 7 |
| *TaGAPC2-6D* Promoter | 3 | 4 | 2 | 1 | 3 | 10 | 8 | 10 |

**Table S2.** Basic local alignment search tool (BLAST) results for potential candidate interacting proteins with TaGAPC2-6D in cDNA wheat library.

| Number | Name | GenBank |
|---|---|---|
| 1 | Plastid glutamine synthetase 2 | ACT22496.1 |
| 2 | Chromatin structure-remodeling complex protein BSH | EMS62932.1 |
| 3 | Ribulose-1,5-bisphosphate carboxylase/oxygenase large subunit | AHI44627.1 |
| 4 | Hypothetical protein TRIUR3-14212 | EMS50462 |
| 5 | Phospholipase Dδ | AK334478.1 |
| 6 | Photosystem II 47 kDa protein | NP_114283.1 |
| 7 | 26S protease regulatory subunit 7 | OAY64297.1 |
| 8 | Hexokinase-2 | AAX84838.1 |
| 9 | Cytochrome b6-f complex iron-sulfur subunit | Q7X9A6.1 |
| 10 | Glutamate receptor | EMT00479.1 |

**Table 3.** All primers used in this experiment.

| **Primers for qRT-PCR** (The underline showed the restriction enzyme sites. F, forward; R, reverse) | | |
|---|---|---|
| Name | Sequences(5′ -3′) | Experiments |
| TaGAPC2–6A F/R | AATCGCATCGCAATCTCG/GCAGCGCAAAGCAAAAAT | qRT-PCR |
| TaGAPC2–6B F/R | AAGTTTTGTATCAATCACCCTGT/ATGTAGGTCATGTAGTCGGTGG | qRT-PCR |
| TaGAPC2–6D F/R | AATCGCATCGCAATCTCG/TGGTGATGAAGGGGTCGT | qRT-PCR |
| **Primers for generating DNA vector** | | |
| Name | Sequences(5′ -3′) | Experiments |
| PR-6A F | CTGCAGGTCGACGGATCCCCGGGCCTTCTCCTTCCAATATGCTT | TaGAPC2-6Apromoter: transient expression |
| PR-6A R | GGTGGACTCCTCTTAGAATTCGGGAAGGAAATCTGGAGCT | TaGAPC2-6A promoter: transient expression |
| PR-6D F | CTGCAGGTCGACGGATCCCCGGGCTTCCAATATGCTTCACCCCG | TaGAPC2-6D promoter:transient expression |
| PR-6D R | GGTGGACTCCTCTTAGAATTCCAGGAAACGAACCCACGGAA | TaGAPC2-6Dpromoter:transient expression |
| Ta1302-F | GGACTCTTGACCATGATGGCTCCGATCAAGATCG | PCAMBIA1302, overexpression |
| Ta1302-R | TCAGATCTACCATGGCCTTGGTGCTGTGCATGTGA | PCAMBIA1302, overexpression |
| Pr1 | GGGGGACTCTTGACCATG | Detection of transgenic Arabidopsis |
| Pr2 | TTACTAGTCAGATCTACCATGGC | Detection of transgenic Arabidopsis |
| Pr3 | AGTTCATCCATGCCATGTGT | Detection of transgenic Arabidopsis |
